# Refractory secondary pneumothorax complicated with lung cancer treated by bronchial occlusion: a case report

**DOI:** 10.1186/s13256-020-02554-y

**Published:** 2020-12-05

**Authors:** Yuto Kato, Miyuki Okuda, Koji Fukuda, Nobuya Tanaka, Seiichi Nobuyama

**Affiliations:** 1 Hirakata Kohsai Hospital, 1-2-1, Fujisakahigashimachi, Hirakata, Osaka 573-0153 Japan; 2 Kansai Medical University Kori Hospital, 8-45, Korihondoricho, Neyagawa, Osaka 572-8551 Japan

**Keywords:** Bronchography, EWS, Refractory pneumothorax

## Abstract

**Background:**

Pneumothorax is defined as the presence of air or gas in the pleural cavity. Secondary pneumothorax usually occurs in patients with overt underlying lung disease, most commonly chronic obstructive pulmonary disease (COPD). Patients with poor lung function often suffer from pneumothorax with a persistent air leak. Various strategies have been employed in the treatment of such refractory pneumothorax. Bronchial occlusion with an Endobronchial Watanabe Spigot (EWS) (Novatech, Grasse, France) has been shown to be useful in treating prolonged bronchopleural fistulas. Although the effects of bronchial occlusion with EWS are known, refractory pneumothorax often involves multiple affected bronchi, and in some cases the affected bronchi cannot be easily identified. In addition, secondary pneumothorax associated with advanced lung cancer often prolongs the treatment of pneumothorax, which can significantly reduce patients’ quality of life and prognosis.

**Case presentation:**

We report a case of refractory pneumothorax where collateral ventilation was successfully treated by bronchial occlusion of the affected bronchi using multiple methods. In August 2019, an 80-year-old Japanese man with asthma and COPD overlap was admitted for exacerbation triggered by respiratory tract infection. During hospitalization, he presented with chest pain due to pneumothorax. Subsequently, a chest drain tube was inserted and pleurodesis was performed; however, the lung could not be sufficiently expanded and an air leak remained. Further investigation revealed a tumor suspicious for lung cancer at the entrance of the left upper lobe bronchus. Due to poor lung function, surgical treatments were deemed high risk. Therefore, we performed bronchial occlusion using the Endobronchial Watanabe Spigot (EWS). Because we could not determine the affected bronchi by computed tomography (CT), we located the affected bronchi by balloon occlusion test and bronchography with iopamidol. After occlusion, the air leak decreased but still persisted. Thus, we performed pleurodesis twice, and the air leak ceased completely.

**Conclusions:**

Refractory secondary pneumothorax, which affected multiple bronchi and developed into collateral ventilation due to lung cancer, was treated successfully with bronchial occlusion and EWS. In cases where the affected bronchi cannot be determined by the balloon occlusion test, bronchography with iopamidol might be an effective treatment.

## Introduction

Patients with poor lung function and underlying diseases such as severe chronic obstructive pulmonary disease (COPD) often suffer from refractory pneumothorax. Various techniques have been employed in the treatment of refractory pneumothorax, such as blood patch, talc pleurodesis, and bronchial occlusion. An Endobronchial Watanabe Spigot (EWS) (Novatech, Grasse, France) is a silicone bronchial blocker developed by Watanabe et al. [1]. Bronchial occlusion with an EWS has been reported to be effective for the management of refractory pneumothorax and thoracic empyema with persistent bronchopleural fistula. This procedure is less invasive and is suitable for patients who are elderly or unfit for surgical procedures [2–4]. Bronchoscopy is required to insert the EWS into a target bronchus. Although the effects of bronchial occlusion using an EWS are known, refractory pneumothorax with severe COPD often involves multiple affected bronchi, and in some cases the affected bronchi cannot be easily identified. In addition, secondary pneumothorax associated with advanced lung cancer often prolongs the treatment of pneumothorax, which can significantly reduce patients’ quality of life and prognosis. We report a case of refractory pneumothorax with collateral ventilation in which the affected bronchi were successfully treated by bronchial occlusion using multiple methods.

## Case presentation

**Fig. 1:**
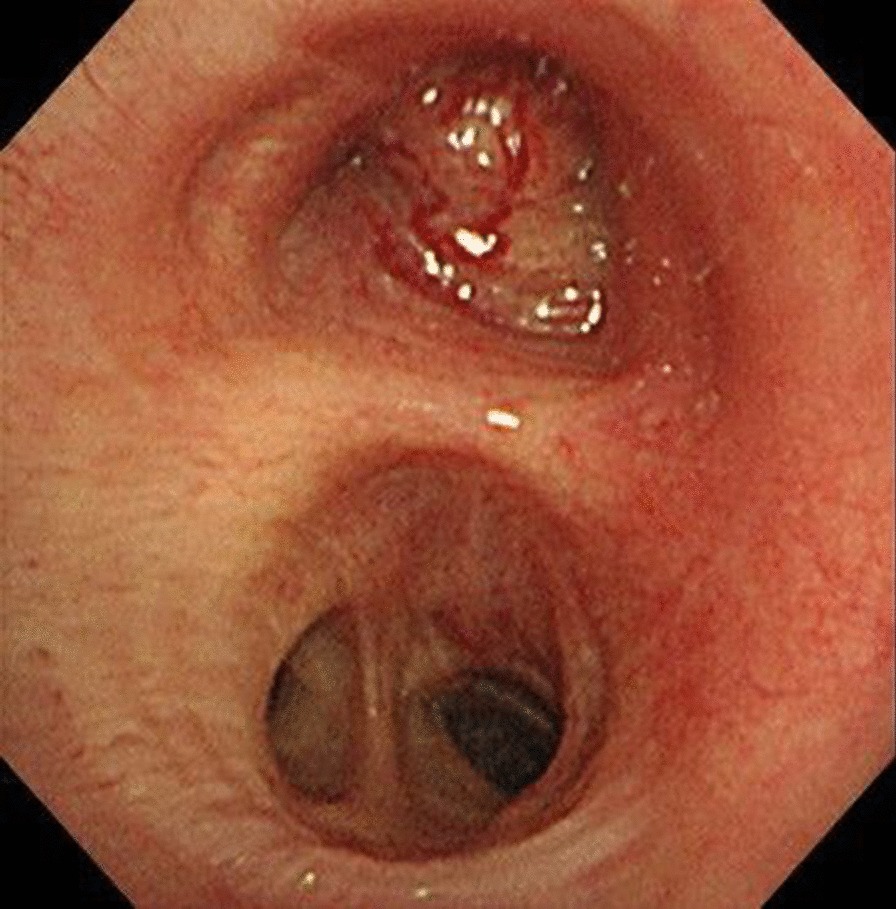
A tumor was observed with a smooth surface at the entrance of the upper left lobe

**Fig. 2:**
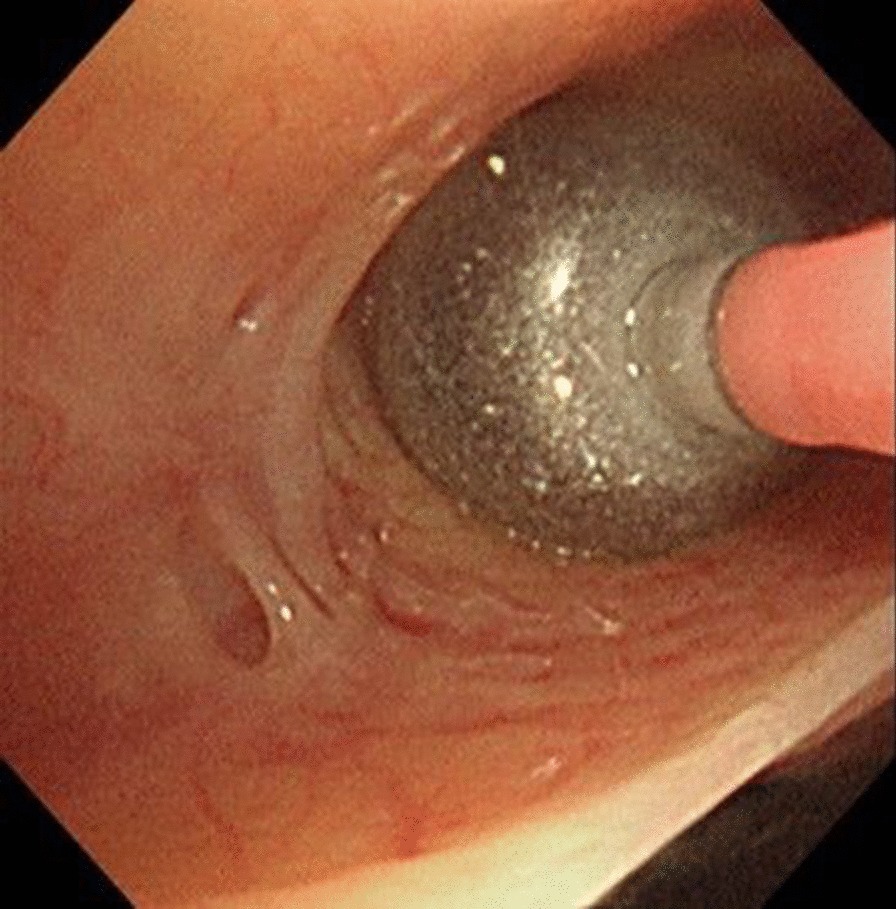
The balloon catheter was inserted into the subsegmental bronchus, and a contrast medium was injected from the tip

**Fig. 3:**
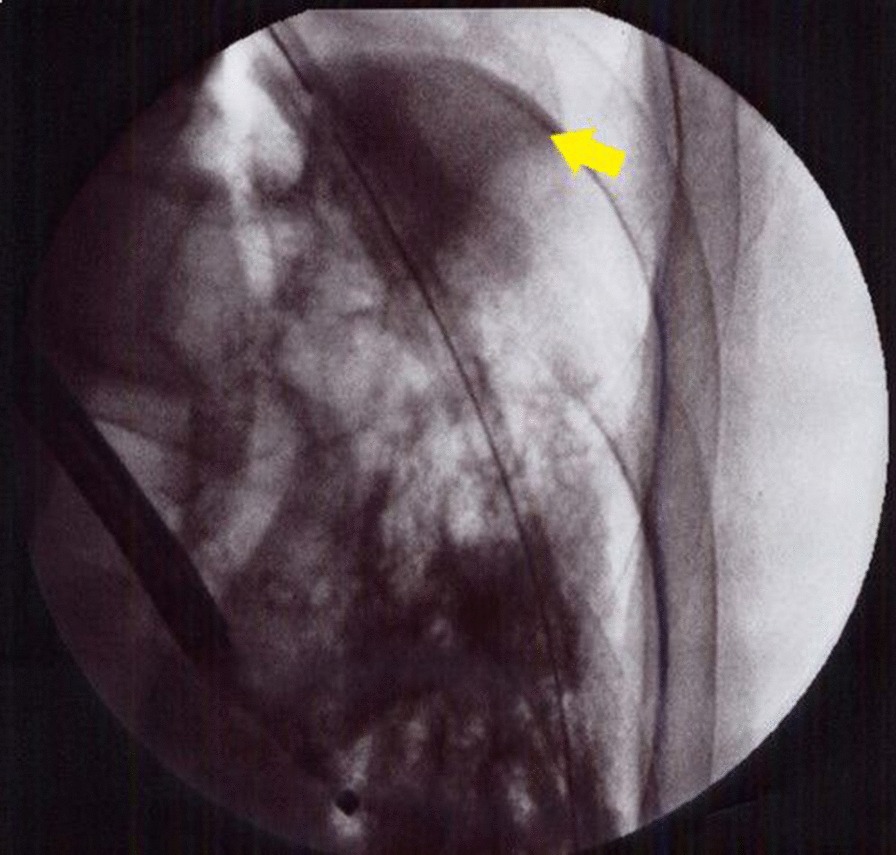
Contrast leakage into the thoracic cavity is indicated by the yellow arrow

An 80-year-old Japanese man hospitalized due to acute exacerbation stemming from asthma and COPD overlap presented with left-side pneumothorax caused by respiratory tract infection. Subsequently, a chest drain tube was inserted; however, a tumor suspicious for lung cancer was present at the entrance of the left upper lobe bronchus. The lung could not be sufficiently expanded, and an air leak was present. We performed pleurodesis twice (first: 200 mL of autologous blood and 4 g of talc; second: 200 mL of autologous blood), but the air leak persisted. Since surgical treatments were considered high risk for this patient due to poor lung function, we decided to treat by bronchial occlusion with EWS. Under bronchoscopy, a tumor was observed with a smooth surface at the entrance of the upper left lobe (Fig. 1). A balloon occlusion test was performed using a balloon catheter (Edwards Lifesciences Corporation, Irvine, CA, USA) with a diameter of 4 Fr and an inflated balloon diameter of 9 mm, to determine the affected bronchi. After the entire left lower lobe bronchus was occluded by the balloon, the air leak ceased. Subsequently, bronchography was performed to identify the affected bronchi at the subsegmental bronchial level. The catheter was inserted into the subsegmental bronchus, and a contrast medium (iopamidol 5 mL and saline 15 mL) was injected from the tip. The contrast medium was injected from the left B8a and left B8b bronchi (Fig. 2). Fluoroscopy confirmed contrast leakage from the left B8b bronchus into the thoracic cavity, and we suspected major leakage at the bronchi leading from the left B8b and the left B6 bronchi (Fig. 3). We then successfully inserted a medium-sized EWS into the left B8b bronchus. Subsequently, we attempted to insert a large-sized EWS into the left B6 bronchus, but the fitting was difficult. Thus, we cut the end of the EWS to size and inserted it into the left B6a bronchus. After EWS insertions, the air leak clearly decreased. Finally, occluding the left B6 and left B8 bronchi by balloon catheter, we confirmed a slight but further decrease in air leakage. Therefore, we judged the remaining air leak to be minor. After bronchial occlusion, we performed pleurodesis twice (first: Picibanil (OK-432) 5 KE; Chugai Pharmaceutical Co. Ltd, Tokyo, Japan, minocycline 300 mg; second: 50% glucose solution and 120 mL of autologous blood), and the air leak ceased completely. The chest drain tube was successfully removed. Several days later, he was diagnosed with pyothorax. The empyema cavity was confined to a small area, and it was difficult to puncture. Therefore, we treated with antibiotics (tazobactam-piperacillin 4.5 g 4 times a day). After 4 weeks of antibiotics therapy, he was transferred to a rehabilitation hospital.

## Discussion

In this case report, we identified two important clinical issues. First, for refractory pneumothorax with collateral ventilation, a combination of balloon occlusion test and bronchography were useful in determining the affected bronchi. Secondly, bronchial occlusion with EWS was effective in treating refractory pneumothorax when pleurodesis seemed ineffective due to insufficient lung dilatation from lung cancer.

It is important to determine the affected bronchi in bronchial occlusion with EWS. Watanabe et al. recommended performing a bronchial occlusion test using balloon catheter to locate the affected bronchi [5]. However, it can be difficult to determine the affected bronchi in patients with advanced emphysema. This is because collateral ventilation develops in the lungs of advanced emphysema patients [6, 7], and if collateral ventilation is present in the affected bronchi, air leaks cannot be completely stopped by occlusion of one of the affected bronchi.

When performing a bronchial occlusion test, the use of a new digital suction device which can quantitatively evaluate the amount of air leakage to determine the affected bronchi is recommended [8, 9]. However, this device was not available for this case.

 Bronchography was used to evaluate the relationship between the bronchi and lesions, but this procedure has rarely been performed clinically since the 1990s [10]. This is largely because noninvasive diagnostic imaging methods, especially computed tomography (CT), have made it possible to visualize the bronchi radiologically, displaying the bronchi as a three-dimensional graphic. However, it is difficult to estimate collateral ventilation prior to bronchial occlusion even with CT. After the balloon occlusion test, the suspected subsegmental bronchus is occluded and a contrast medium is injected from the tip of the catheter. Occasional contrast flow that is clearly different from bronchial branches can be observed under fluoroscopy. If the contrast medium selectively injected into the bronchus flows into the thoracic cavity under fluoroscopy, the selected bronchus is considered to be affected. This technique has been reported as particularly effective in cases where multiple affected bronchi are present [11]. In our case, for safety purposes, an iodine-based nonionic angiographic contrast medium, iopamidol, diluted with saline was used. Iopamidol has been established as a safe angiographic contrast medium, while conventional bronchial contrast media have been discontinued and are no longer used [12, 13]. It has been reported that bronchography can be performed at a lower cost than CT; however, to our knowledge, there are no reports in which the affected bronchi were determined by bronchography. Although pneumothorax is rarely associated with lung cancer, its treatment has not been sufficiently studied to date.

In recent years, EWS has been made available for refractory pneumothorax. Although many successful treatments have been reported, most were benign diseases such as COPD and empyema. According to a previous study using EWS for air leaks associated with lung cancer, the number of EWS occluded and the period from onset of pneumothorax to bronchial occlusion with EWS was the same as with other diseases. Furthermore, successful chest drain tube removal was achieved in about 80% of cases, using both bronchial occlusion with EWS and pleurodesis [14]. The removal of the drainage tube is advantageous, even in terminally ill patients.

## Conclusions

We report a case of refractory secondary pneumothorax in which collateral ventilation developed due to lung cancer. We found that bronchial occlusion with EWS was effective for this case with multiple affected bronchi. Therefore, in cases where the affected bronchi cannot be determined by the balloon occlusion test, bronchography with iopamidol might be effective.

## Data Availability

The authors declare that the data supporting the findings of this case report are available within the article and its supplementary information files.

## Abbreviations

COPD: Chronic obstructive pulmonary disease
EWS: Endobronchial Watanabe Spigot
CT: Computed tomography
